# Outburst floods provide erodability estimates consistent with long-term landscape evolution

**DOI:** 10.1038/s41598-018-28981-y

**Published:** 2018-07-12

**Authors:** Daniel Garcia-Castellanos, Jim E. O’Connor

**Affiliations:** 1 Instituto de Ciencias de la Tierra Jaume Almera, ICTJA-CSIC, Solé i Sabarís s/n, 08028 Barcelona, Spain; 2 U.S. Geological Survey, 2130 SW Fifth Ave., Portland, OR 97201 USA

## Abstract

Most current models for the landscape evolution over geological timescales are based on semi-empirical laws that consider riverbed incision proportional to rock erodability (dependent on lithology) and to the work performed by water flow (stream power). However, the erodability values obtained from these models are entangled with poorly known conditions of past climate and streamflow. Here we use the erosion reported for 82 outburst floods triggered by overtopping lakes as a way to estimate the outlet erodability. This avoids the common assumptions regarding past hydrology because water discharge from overtopping floods is often well constrained from geomorphological evidence along the spillway. This novel methodology yields values of erodability that show a quantitative relation to lithology similar to previous river erosion analyses, expanding the range of hydrological and temporal scales of fluvial incision models and suggesting some consistency between the mathematical formulations of long-term and catastrophic erosional mechanisms. Our results also clarify conditions leading to the runaway erosion responsible for outburst floods triggered by overtopping lakes.

## Introduction

Recent quantitative understanding of river erosion over geological timescales is based chiefly on semi-empirical laws that relate fluvial incision to stream power or shear stress and to rock resistance (erodability)^1–4^. Although such laws are foundational to studies of landscape evolution, their application is limited because most long-term empirical observations of river incision cannot separate the effects of channel resistance to erosion from that of the past hydrological conditions, specifically the magnitude and variation of geomorphically effective discharge^1,5–7^. For example, Lavé & Avouac (2001; ref.^8^) provided values for erodability (Supplementary Table 2) by obtaining incision rates from river terrace dating and by estimating an averaged basal shear stress at Himalayan rivers, relying on an arbitrary choice of the critical flood intensity that triggers bedrock erosion. The prediction power of the erosion laws is thus hindered by the scant knowledge of the past hydrological conditions, in addition to the limitations in measuring river incision. Consequently, the determinations of *erodability*, the rock-intrinsic parameter that relates water flow to erosion rate (Eq. 4 in the *Methods*), are in most situations entangled with the uncertainties in the quantification of the history and the variability of water discharge and climate^9–11^. The erodability values thus obtained remain linked to the arbitrary adoption of average or effective discharges. Furthermore, surface exposure dating in Icelandic rivers^12^ and river-transport numerical models^13^ suggest that the uneven temporal distribution of water flow strongly influences the entrenchment of river channels. In the Tsangpo Gorge in the eastern Himalaya, a singular outburst flood from a catastrophically drained lake may have accounted for downstream valley erosion equivalent to 1–4 ky of normal flow conditions^14^. Such factors challenge the adoption of arbitrary effective discharges for river incision modelling.

Here we describe a distinct method to evaluate rock erodability under well-constrained hydraulic conditions. Outburst floods triggered by breaching of rock-bound water-filled basins have produced some of Earth’s largest known floods as well as spectacular erosional landscapes^15,16^. These floods result from lakes overflowing and rapidly eroding the outlet. Their water discharge evolution (hydrograph) can be constrained from measures of the peak discharge in combination with the geometry of the outlet and the source lake. Such floods emanate from diverse natural settings, including overtopped volcanic calderas^17^, tectonic basins like Lake Bonneville^16,18–20^, ice-dammed lakes, and even crater lakes on Mars^21^. Their peak discharge is derived from geomorphological evidence within the spillway and downstream. Historical floods, particularly those from constructed dam and landslide dam failures, are well studied for hazard assessment and they allow a direct measure of the peak discharge^22,23^. Compilations of such studies, including also dam breach experiments, provide empirical relationships between lake volume, dam height, and peak discharge, albeit with a scatter of several orders of magnitude in discharge^15^. Even though much of this scatter is likely related to variation in outlet rock strength^24–26^, most dam-breaching and lake-overtopping models adopt an instantaneous dam removal or a constant erosion rate to estimate outflow hydrographs^19,27,28^ to avoid specifically addressing the complex breach erosion processes. Incorporating erosion laws to the models of dam-breach floods should therefore provide a quantitative link between peak discharge and erodability.

The erosion of large intramountain sedimentary basins is often triggered by overtopping lakes and has been revealed also as key mechanism controlling long-term landscape evolution and large drainage changes. Notable examples include the Ebro Basin^29^ (NE Spain), the Colorado Plateau^30^, the Sichuan Basin^31^, the Dover Strait^32^, and the Zagros Mountains^33^. When endorheic (closed-drainage) basins dig outlets, the regional base level falls and a substantial part of the sedimentary basin is quickly excavated. Applying long-term river incision models to lake overtopping scenarios may therefore bring new light on the mechanics of large basin captures that we now know are frequent and an important mechanism in landscape evolution. Thus, the questions we address in this study are: From a mechanistic point of view, what can we learn from outburst floods regarding fluvial erodability and long-term river and relief evolution? And conversely: do landscape evolution and river erosion models offer insight into the conditions conducive to outburst floods? We hypothesize that the basal shear-stress model of fluvial erosion (see Methods), a widely used approach for modelling long-term landscape evolution, is applicable to the erosion of the sill during outburst floods and that it can provide reliable estimates of outlet erodability.

## Results

**Figure 1:**
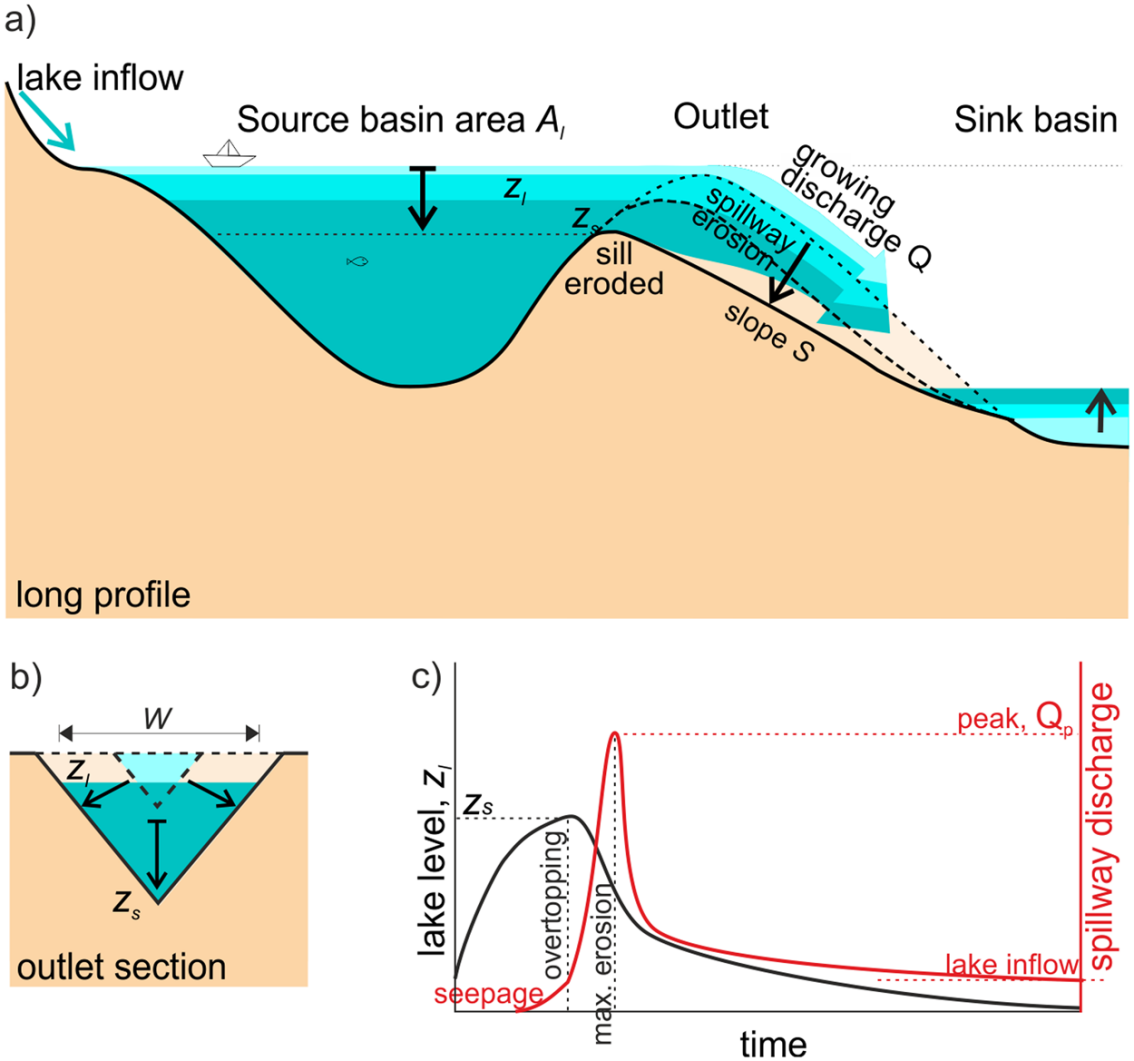
Conceptual model of lake overtopping and outburst flood development as a result of interactions among outlet erosion, water discharge, and lake drawdown. For the parameters involved see the Methods. (**a**) Section along the outlet. (**b**) Section across the outlet. (**c**) Schematic evolution of lake level and outflow discharge before and after overtopping.

To test this hypothesis, we model peak discharge measures from 82 outburst floods^15,16^ (Supplementary Table 1) ranging from laboratory experiments to large paleolake overtopping events. We formulate a simple model of the lake outlet sill (Fig. 1) accounting for the feedback between outlet erosion and water discharge from the overtopping lake (see *Methods*). Erosion rate is calculated as the product of erodability (assumed to be controlled by the lithology) and the shear stress at the base of the flow. Since the water is coming from a lake, in-transit bedload is negligible and we assume it has little influence on erosion rate. This allows adapting the stream power-law formulation, which is common in river incision models intended to describe the long-term evolution of river profiles. The flood simulations respond to the enlargement and deepening of the outlet due to erosion in conjunction with the gradual drainage and level lowering of the lake. The fall of the lake level is calculated as a function of the hypsometry (area-elevation relationship) of the lake floor. Thus, for each documented lake-overtopping flood (Supplementary Table 1), we search the erodability *k*_*e*_ of the outlet that best fits the peak discharge *Q*_*p*_ derived from previous geomorphological studies or experimental settings.

### Forward numerical model

**Figure 2:**
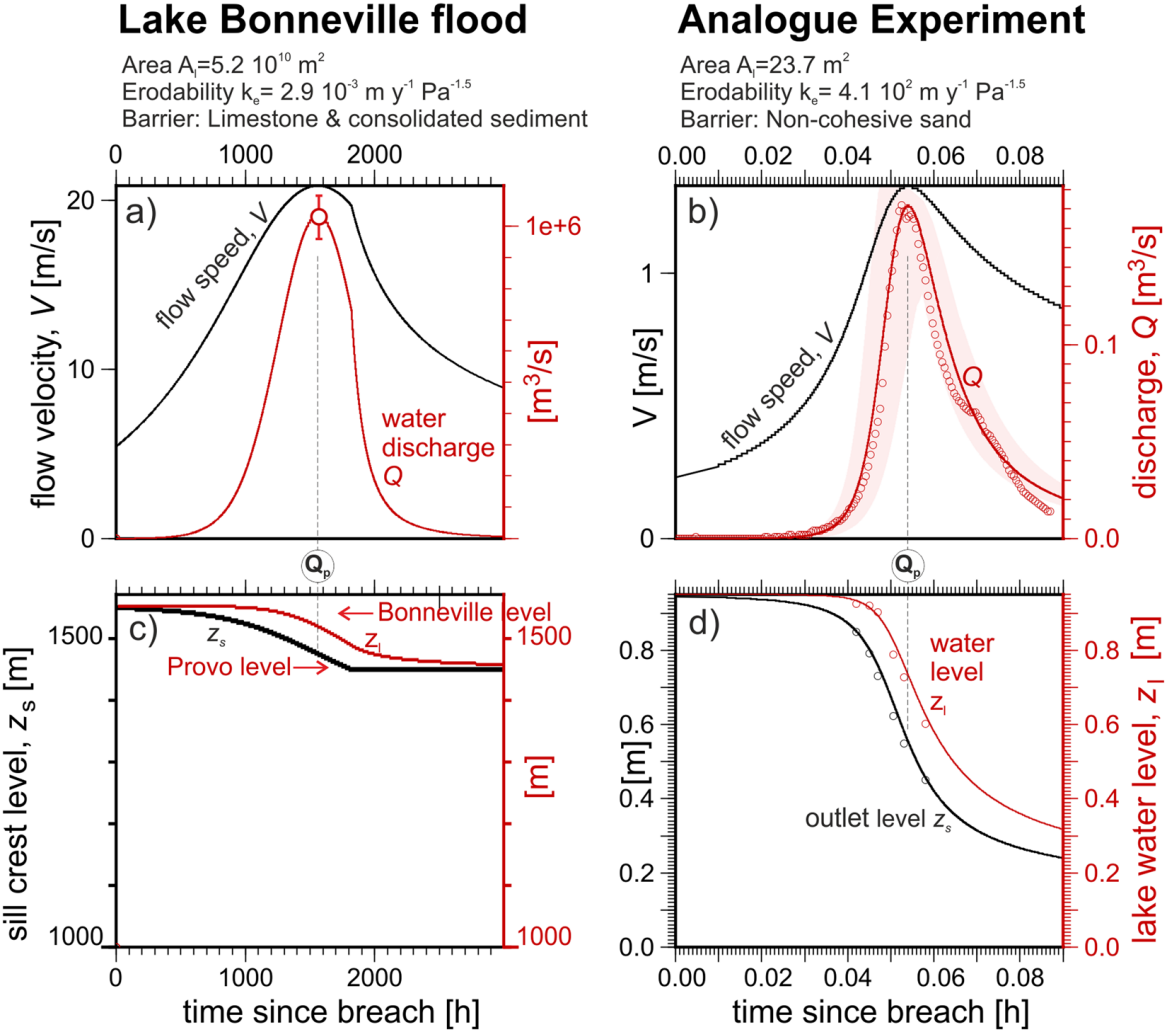
Evolution of two model runs inspired by the Lake Bonneville megaflood (left) and an analogue physical experiment^25^ (right). Model results: water velocity and discharge (**a**,**b**) and the levels of the lake water and the outlet sill (**c**,**d**); the area shaded in pink shows the effect of a 10% change in erodability; circles indicate field and experimental measurements of flow. Erosion in the Bonneville setting is stopped when reaching the Provo lake level, to account for the harder Paleozoic limestone underlying the original fluvial sediment barrier^20^. Note the initial exponential increase in discharge in both settings, caused by the feedback between outlet erosion and flow through the outlet (see *Methods*).

To solve the forward numerical model (Eqs 1 to 6 in the *Methods*) we developed a simple code named *spillover* (written in C, available on gitHub). Figure 2 shows the results of applying this model to two sample scenarios. First, we model the overtopping of Pleistocene Lake Bonneville, a large lake with an area of 5.2 10^10^ m^2^ that triggered one of the largest documented outburst floods when it overtopped a 100-m tall barrier made of Quaternary and Tertiary sediment at Red Rock Pass, Utah (see Supplementary Table 1). The flood incised approximately 120 m at the outlet, attaining a peak discharge of *Q*_*p*_ = 10^6^ m^3^ s^−1^, consistent with estimations from hydraulic modelling of downstream high-water evidence^16^. The Lake Bonneville basin is set up with a box-like hypsometry from the Bonneville level (1552 m) down to the Provo level (1440 m). Once the outlet thalweg reached the Provo level, we halt incision to simulate the effect of the basement rock at Red Rock Pass^34^. The initial lake level is arbitrarily set to 1 m above the sill level, but this parameter barely affects the peak discharge. The observed *Q*_*p*_ is fit by the model (Fig. 2a) for an erodability *k*_*e*_ of 2.9 10^−3^ m yr^−1^ Pa^−1.5^, a value that following our hypothesis should represent the lithology eroded at the Red Rock Pass outlet: consolidated fluvial sediment lying on a much harder basement of Paleozoic limestone. As a second example, we simulate (Fig. 2b) the 2012 analogue Experiment 4 by Walder *et al*.^25^ where a 1-m-high compacted-sand barrier impounded a 23.7 m^2^ lake. We use an hypsometric curve that linearly reduces the water surface to 0 m^2^ when the lake is empty (at *z* = 0 m). The initial water level is at z = 1.005 m (0.5 cm above the sill level). The measured hydrograph in Fig. 2b is now satisfactorily fit with a much higher erodability *k*_*e*_ of 4.1 10^2^ m yr^−1^ Pa^−1.5^, in agreement with the weaker nature of the impounding dam relative to Bonneville’s.

### Analytical model

**Figure 3:**
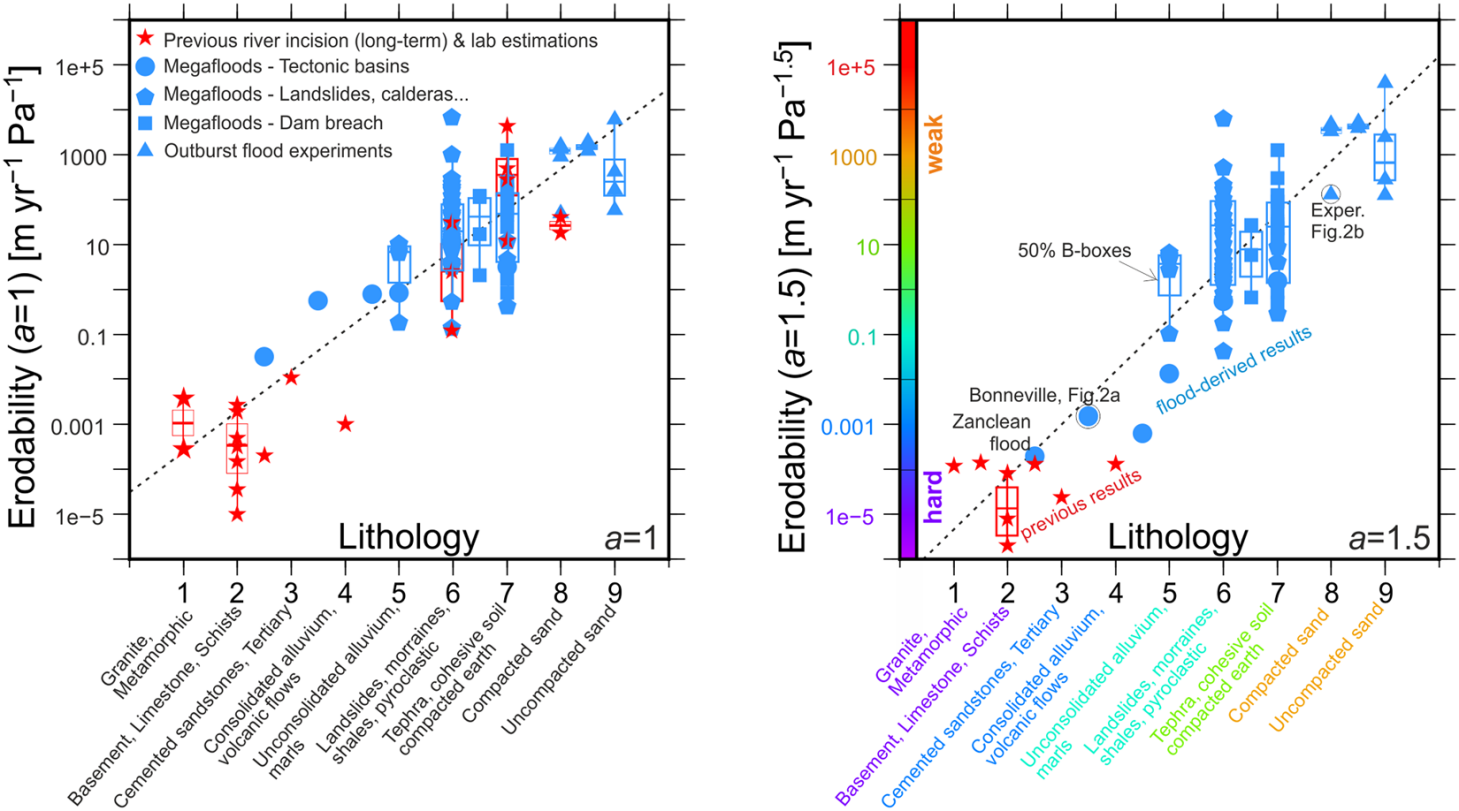
Erodability estimated from outburst floods from overtopping lakes as a function of barrier type (blue symbols). The newly calculated erodability is based on the measured flood peak discharges using analytical solutions corresponding to exponents in the erosion law of *a* = 1 (Eq. 17 in the *Methods*; panel (a); and *a* = 1.5 (Eq. 9, panel b; same legend as panel (a). The results from outburst floods are directly comparable to fluvial erodability estimates from long-term river studies (red; Supplementary Table 2) available for each *a* value. The calculated erodability values span up to two orders of magnitude (indicated by the B-boxes encompassing 50% of estimates for each lithology class), likely due to factors other than lithology not explicitly accounted for here, such as the grain size of non-cohesive materials or fracturing for bedrock. However, the overall distributions of outburst flood and river incision determinations of erodability follow mutually consistent trends (dashed lines show the logarithmic regression of all data, with a correlation coefficient of 0.86 for *a* = 1.0 and 0.87 for *a* = 1.5). Red symbols correspond to published estimations of erodability. Blue symbols are new estimations based on our method. The colour code in the axis of the right plot is only to link with Fig. 4.

We now calculate the outlet erodability for the 82 lake breaches for which peak discharges were independently estimated^16^ (Fig. 3) using Eq. 9 (see *Methods*). The lakes considered have volumes spanning 16 orders of magnitude, and include overtopped tectonic basins, volcanic calderas, landslide-dammed lakes, and experimental dam failures. For simplicity and reproducibility, we calculate erodability using the analytical solutions for the peak discharge (Eqs 9 and 17, *Methods*) instead of the forward model used in Fig. 2. Figure 3 shows the resulting *k*_*e*_ for two values of the exponent of the erosion law, *a* = 1.0 and *a* = 1.5. Separately, we have qualitatively classified the lithology of their outlet rock and assigned an index (Supplementary Table 1; Fig. 3) ranging from 1 (hardest: metamorphic and granitic rocks) to 9 (softest: non-cohesive uncompacted sand), following previous measures of rock strength^35,36^. In some cases, we straddle two classes because of heterogeneous barriers. For example, experimental dams combining sand and pebbles have been assigned an index value of 6.5 (between 6 for landslides and 7 for overcompacted earth).

The erodabilities estimated from the 82 outburst floods are consistent with their lithologic classification as well as with values determined from previous measurements of river incision (red stars in Fig. 3; references in Supplementary Table 2). When comparing the results (Fig. 3) to the former independent estimates of peak discharge, one must take into account the uncertainty in the width parameter *k*_*w*_ (which may change during the course of the flood), the uncertainty in the Chezy constant *C*_*z*_, and the significant uncertainty inherent to the measurements of outburst flood discharge that are used as input data for our estimates. The combined outburst flood and river incision erodability measurements give a logarithmic trend of *k*_*e*_ as a function of the lithological index (Fig. 3) for the two power values considered: *a* = 1.0 and *a* = 1.5, highlighting the strong control of rock type on water erosion rates. The similar trend of the two data sets shows that the method for modelling outburst flood erosion during singular outburst floods provides a good first-order measure of erodability and that the resulting values are consistent with those obtained from river incision measurements. The outburst flood measurements, however, extend the range of observed lithology types and erodability values, and are for well-constrained hydraulic conditions compared to the hydraulic assumptions for most river incision studies.

## Discussion

Our approach relies on the shear-stress model of incision, which is a simplistic approach to the primary channel-incision processes of entrainment, plucking, abrasion, and cavitation^11,37^. A key difference for outburst floods is that outlet erosion during basin-breach floods involves essentially clear water exiting the lake, in contrast to sediment-laden floods in more typical river environments. Consequently outlet incision by sediment abrasion —as called upon for much river incision^9,38^—is not likely effective during large outburst floods. Outburst floods clearly do erode outlets, so entrainment (for outlets composed of unconsolidated materials), plucking^2^ and possibly cavitation^37,39^ (for outlets of harder materials) are important erosion processes. Whereas bedrock erosion by abrasion is strongly dependent on sediment transport rates and less so on flow hydraulics^40^, erosion by entrainment, plucking and cavitation is strongly dependent on hydraulics applied shear stress. The overlap and similar trends of the erodability values from river incision measurements and those from the singular clear-water outburst floods (Fig. 3), together with recent studies in mountain fluvial environments^2,41^, may indicate that plucking is a more important process than generally recognized for a broader range of timescales from hours to millions of years. However, we cannot exclude that this overlap is simply reflecting a similar mathematical dependence on water flow governing both mechanisms. Discriminating the relative importance of plucking vs. abrasion is beyond the scope of this study, but we show that sill erodability strongly influences the magnitude of outburst floods from breached lake basins, and that future models that explicitly implement more mechanistic approaches to each individual process may allow separating the relative importance of plucking and abrasion.

The results also show that analysis of singular outburst floods with known peak discharges from breached lake basins provide another means for estimating rock erodability. Figure 3 shows that, despite avoiding the uncertainty of past hydrological conditions, our approach still results in uncertainties spanning two orders of magnitude in erodability estimates. This indicates uncertainties derive from other factors as well. Possible sources of error are the indirect determination of the peak discharge or the simplifications adopted for the analytical model. The latter may be avoided by future specific forward modelling of each flood separately. However, within these uncertainties the estimated values are quantitatively compatible with those previously derived from long-term fluvial incision studies. Remarkably, the adopted shear-stress model adequately characterizes outlet erosion for the relatively sediment-free incision of lake outlets, yielding erodability values that are quantitatively close to those obtained from river incision observations and indicating a wide applicability to fluvial incision.

**Figure 4:**
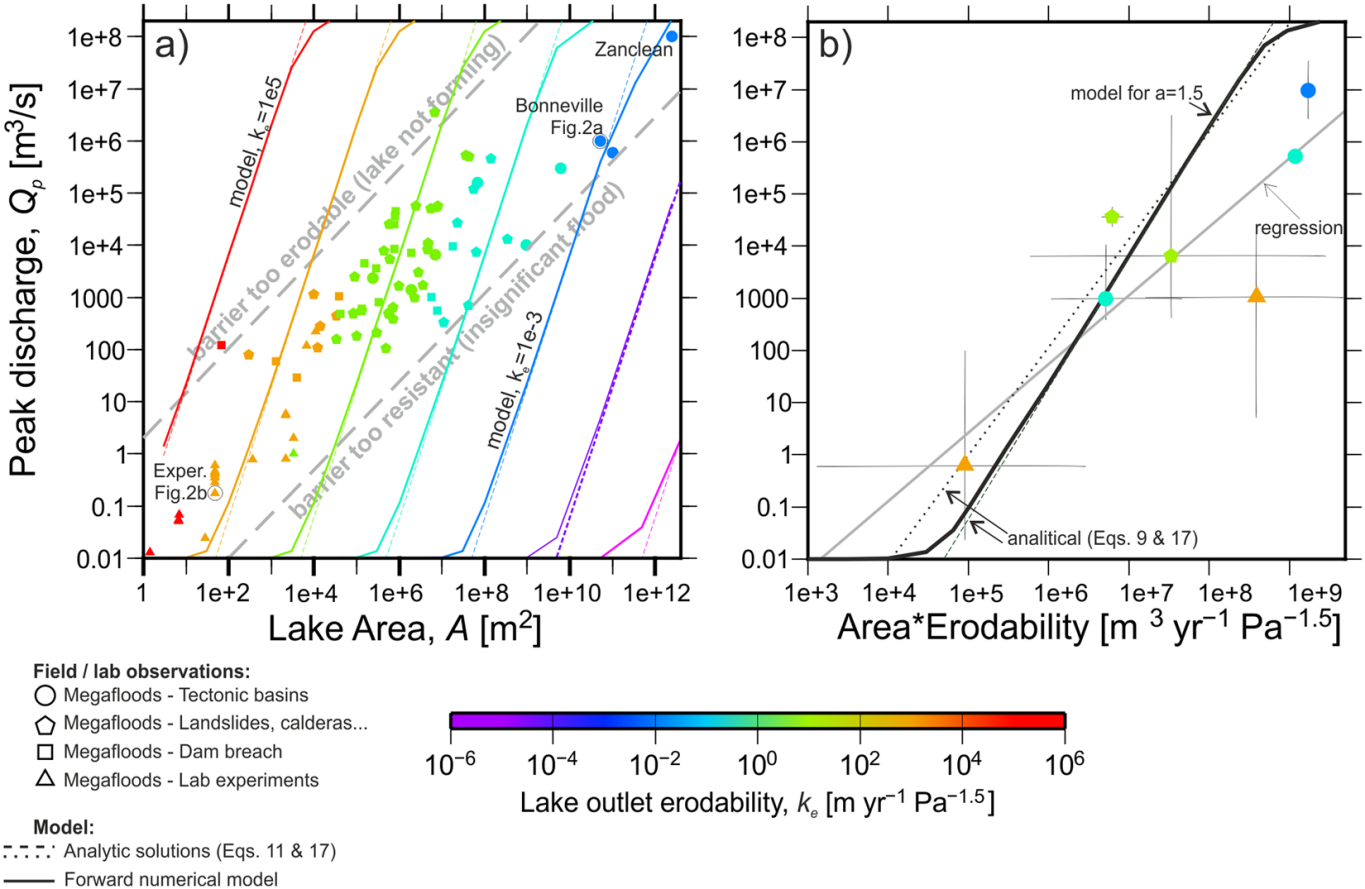
Peak discharges from overtopping floods: data and model results. (**a**) Peak discharge as a function of the area of the source lake *A*. Measured flood discharges (symbols; Supplementary Table 1) are compared to the forward model results (plain lines) and the analytic solution (Eq. 9; dashed lines). Symbols are coloured according to the erodability obtained from Eq. 9. The observed floods are restricted to a *Q*_*p*_-*A* band indicated by the two thick, grey-dashed lines. The model results, however, show that the positive feedback between erosion and water discharge could a priori take place outside of that space, along the lines shown for constant erodability values. Their absence indicates other controls, which we interpret as due to undetected modest floods from large basins (lower-right part of the plot) or because the barriers are too weak to hold the deep water reservoirs required to produce large floods for relatively small lake areas (upper-left part of the plot). Symbol code as in Fig. 3. (**b**) Comparison of the relation between peak discharge and the product of lake area and outlet erodability as predicted by Eq. 9, the analytical formulation, and the observations (coloured symbols). Each symbol corresponds to an average for a lithological class of log(A*k_*e*_) and *log(Q*_*p*_). The erodability color code is here based on the correlation with lithology found in Fig. 3. The discharge of the Zanclean flood of the Mediterranean^19^ is model-based and shown for reference only.

Documented outburst floods, evaluated by this methodology, also indicate conditions favourable for basin breaching. Specifically, outburst floods (symbols in Fig. 4a) are restricted to a relatively narrow domain in the area-discharge plot, ranging from small experimental lakes with very erodible outlets (sand) to large lakes with harder outlets (bedrock). In contrast, the model predicts (lines in Fig. 4a) a much wider plausible range of lake area/erodability combinations. For example, the model predicts floods comparable in discharge to those from breached landslide lakes (10^2^–10^6^ m^3^/s) for larger lakes with harder outlets (domain below the lower dashed line in Fig. 4a), but we find no such instances in the geological record. The absence of such floods is likely because larger basins evolve over many millions of years and the geological record of incision of such relatively modest floods has been masked or obliterated by erosion. Additionally, outburst floods from large tectonic basins are only associated with erodability values *k*_*e*_ as low as ~10^−4^ m yr^−1^ Pa^−1.5^, mostly associated with cemented or consolidated clastic rocks (Fig. 3b). Harder lithologies such as granite or metamorphic rocks may generally erode too slowly to accomplish Eq. 1 and trigger catastrophic discharges from runaway outlet erosion. This is also consistent with the near-absence of floods from breached lava-flow dams^16^, compared to other natural dams such as landslides and glacial moraines creating lakes of similar area (Supplementary Table 1). Nevertheless, the erosion recorded at the granitic foot of the 57-km2 Ricobayo reservoir in Spain^42^ indicates that outburst floods at harder lithologies cannot be excluded. Also predicted by the model but absent from the observational record are large floods from lakes of smaller surface area and highly erodible outlets (region above the upper dashed line in Fig. 4a). We infer that weak barriers, such as those made out of unconsolidated sand, are prone to failure before sufficient water is impounded to depths necessary for producing large floods. For example, water volume and dam height in experiments with sand barriers (Supplementary Table 1, Fig. 2b) are limited by the technical capability to fill impounded basins fast enough before sapping dismantles the barrier^25^. These two end-member scenarios involving very hard and very weak barriers likely limit the range of documented outburst floods from eroding basin sills, leaving documented outburst floods to be mostly in the parameter space between the two dashed lines in Fig. 4a.

Despite the satisfactory correlation between erodability and lithology, the applicability of our method to risk assessment is limited at this point. Observations of peak discharge vary by more than 2 orders of magnitude for a given combination of lake area *A* and outlet erodability *k*_*e*_ (see the 50% box plot in Fig. 4b). While the model captures the strong correlation between peak discharge and A*k_*e*_, a better assessment of outburst floods from overtopping lakes will require better understanding and implementation of erosion processes^26^ that quantitatively account for sedimentary characteristics for non-cohesive barriers and rock properties for bedrock outlets. Such analyses, besides improving hazard assessment related to dam failure, may also provide for better mechanistic erosion models^11^. The results here presented encourage and provide a means for incorporating the large flooding events as an integral part of models for the long-term evolution of topography.

## Methods

We develop a simple 0D formulation focused on the lake’s outlet sill, which in most cases controls the water discharge after overtopping. While 2D and 3D breach flood modelling has advanced significantly over the last decade^43^, we aim here at an easily reproducible algorithm that can be systematically applied to a large set of overtopping outburst floods. Similar to recent models of dam breaching^19,27^, we calculate the erosion of the sill as proportional to shear stress at the base of the flow, although here the erosion formulation is directly related to the erodability of the substrate (separated from the hydrological component) and water velocity is calculated based on a critical-flow approach applied at the sill^19^, instead of the outflow channel. To allow a direct comparison with independent river incision studies, we elaborate a new formulation that is consistent with long-term fluvial erosion models.

Consider a lake at level *z*_*l*_ spilling through an outlet consisting of a sill with an average elevation *z*_*s*_ (*z*_*l*_ > *z*_*s*_) at a reference time *t* = 0. A necessary condition for the initiation of an overtopping outburst flood is that the rate of erosion from the sill must be faster than the rate of lake level decrease:

1 $$\frac{d{z}_{s}}{dt} < \frac{d{z}_{l}}{dt}$$

Note that *z* is positive upwards and therefore both sides of the equation are negative. The rate of lake level decrease is determined from the continuity equation

2 $$\frac{d{z}_{l}}{dt}=-\,\frac{Q}{A}$$

where *A* is the lake area and *Q* is the water discharge (m^3^ s^−1^), both being time-dependent. The effect of climatic parameters is negligible because outburst floods almost always involve water discharges much larger than those produced by normal meteorological conditions. A water mass balance allows obtaining *Q* from the outlet width *W*, the mean flow velocity *V*, and the mean flow depth:

3 $$Q=W\,({z}_{l}-{z}_{s})V$$

To formulate the erosion rate at the outlet sill of the lake we adopt the shear stress model for river incision. Although the mechanics of fluvial erosion are complex and the relative importance of processes as plucking, abrasion, and cavitation is currently disputed^41,44^, a power law function of the basal shear stress *τ* at the river bed has been used extensively to model the first order controls on erosion^45^. Here we assume that this holds true for lake outlets during overtopping outburst floods:

4 $$\,\begin{array}{cc}\frac{d{z}_{s}}{dt}=-\,{k}_{e}{(\tau -{\tau }_{c})}^{a} & (\tau  > {\tau }_{c})\end{array}$$

with *dz*_*s*_*/dt* = 0 if *τ* < *τ*_*c*_, with *τ*_*c*_ being the critical shear stress needed for erosion. Note that the competing blanketing and abrasive effects of bedload sediment, usually relevant in river incision^9^, can be neglected at lake outlets, where water is relatively free of sediment bedload. Another assumption implicit in this formulation is that the erodability *k*_*e*_ is an intrinsic property of a given rock lithology, while processes such as alteration or fracturing can significantly affect rock strength. For *a*, we consider values of 1 and 1.5 (ref.^7^). While higher exponent *a* values may be more appropriate for cavitation, we do not consider such values because of the absence of comparative independent estimations of *k*_*e*_. Because the sill is close to critical flow, cavitation is only likely for the largest outburst floods where flow depth is larger than a few meters^37^. Therefore, we do not specifically incorporate cavitation to the erosion formulation.

The sill elevation is regulated by the erosion rates not only at the sill itself but also along the outlet channel downstream. The critical flow across the sill is the main hydraulic control on the discharge from an overtopping lake^25^, whereas along the spillway it is slope that controls the shear stress^19^. Consequently, we consider erosion of both the sill and the downstream outlet channel.

### Sill-focused formulation

The basal shear stress at the sill can be calculated using the Darcy-Weisbach equation^46^:

5 $$\tau =\frac{\rho g}{{C}_{z}^{2}}{V}^{2}$$

where *C*_*z*_ is the Chezy constant ranges typically from 35 to 70 m^1/2^ s^−1^ (ref.^47^), ρ is the water density, and *g* the acceleration of gravity. The average water flow velocity above the sill *V*, can be calculated applying the critical flow condition at the sill, where the flow changes from subcritical to supercritical:

6 $$V=\sqrt{g({z}_{l}-{z}_{s})}$$

Adopting *a* = 1.5 and *τ* ≫ *τ*_***c***_, Eq. 4 can now be rewritten as

7 $$\frac{d{z}_{s}}{dt}=-\,\frac{{\rho }^{3/2}{g}^{3}}{{C}_{z}^{3}}{k}_{e}{({z}_{l}-{z}_{s})}^{3/2}$$

Consider the channel width proportional to flow depth (preservation of the cross-sectional shape of the outlet)

8 $$W={k}_{w}({z}_{l}-{z}_{s})$$

where *k*_*w*_ is a unitless proportionality constant. Combining Eqs 3, 6–8, the condition in Eq. 1 leads to peak discharge *Q*_*p*_ taking the form

9 $${Q}_{p}={(\frac{{\rho }^{5/2}{g}^{9/2}}{{C}_{z}^{5}{k}_{w}})}^{3/2}{k}_{e}^{5/2}\,{A}^{5/2}\,{\rm{.}}$$

This gives an estimation of the peak discharge as a function of fixed parameters, except for the lake area *A*, for which the initial value is taken as a first approach for the analytical results shown in Fig. 4.

To obtain an analytical solution of the feedback between outlet erosion and discharge, we can make use of the Manning’s roughness coefficient *n* as a function of the Chezy coefficient and the hydraulic radius *R*_*h*_ (measured in m) of the sill, a measure of its flow efficiency:

10 $$n=\frac{{R}_{h}^{1/6}}{{C}_{z}}$$

For a channel substantially wider than deep, ${R}_{h}\approx ({z}_{l}-{z}_{s})$, and now Eq. 7 can be rewritten as

11 $$\frac{d{z}_{s}}{dt}=-\,{\rho }^{3/2}{g}^{3}{n}^{3}{k}_{e}({z}_{l}-{z}_{s})$$

implying that the sill is initially eroded at exponentially-increasing rates as long as *z*_*l*_ does not drop significantly. For an *n* value of 0.045 (appropriate for natural channels), the characteristic time for this exponential increase is about *3*.*4* *e*^*−4*^*/k*_*e*_ (in years if *k*_*e*_ is given in m yr^−1^ Pa^−1.5^). Using the range of *k*_*e*_ values compiled in Supplementary Table 2 (*a* = 1.5), water discharge during the initial stages of overtopping increases by a factor *e* every 3 to 200 yr for hard cohesive rocks ranging from flysch to limestone, and in the order of seconds to minutes for non-cohesive sand. The former are values consistent with the time scales proposed for the Zanclean flood across the Strait of Gibraltar^19^; the latter satisfactorily match the experimental settings of sand dams in Supplementary Table 1, respectively. This initial exponential growth is limited by the reduction of the lake’s area as its level drops, depending on the lake’s hypsometry, and the coincident reduction in head at the outlet.

### Spillway-focused formulation

The erosion at the sill can be affected by the erosion rates downstream along the outlet channel, since such erosion may propagate upstream by erosional retreat. This effect can be addressed more appropriately by formulating the erosion along the spillway channel, aiming at an expression for the peak discharge dependent on the spillway slope. Shear stress *τ* at the spillway can be here approached as in mountain rivers or constructed channels, as the product of *ρg*, the mean water column depth, and the channel slope *S*:

12 $$\tau =\rho g({z}_{l}-{z}_{s})S$$

This formula implicitly assumes a steady flow, although the error induced is negligible in comparison with the orders of magnitude of scatter in the peak discharge data. For *τ* ≫ *τ*_***c***_, Eqs 4 and 12 lead to an expression for the erosion rate^19^ similar to Eq. 11 but involving the slope of the spillway:

13 $$\frac{d{z}_{s}}{dt}=-\,{(\rho gS)}^{a}{k}_{e}{({z}_{l}-{z}_{s})}^{a}$$

As in the earlier sill-focused approach, erosion rate increases exponentially in timescales of 1 to 100 years for the hardest rocks listed in Supplementary Table 2, and 3 to 30 seconds for non-cohesive sand. To calculate the water flow along the spillway we apply the empirical Manning’s relationship between velocity *V* and the hydraulic gradient *S*:

14 $${\rm{V}}=\frac{1}{n}{({z}_{l}-{z}_{s})}^{\tfrac{2}{3}}{S}^{\tfrac{1}{2}}$$

where *V* is the average velocity (m s^−1^), and *n* = *0*.*035* s m^−1/3^ is the roughness coefficient. Combining Eqs 3, 8, 12–14, an expression is obtained for erosion of the sill equivalent to a stream power law^48^:

15 $$\frac{d{z}_{s}}{dt}={K\text{'}Q}^{m\text{'}}\,{S}^{n\text{'}}$$

16 $${\rm{K}}\text{'}={k}_{e}{(\rho g)}^{a}{(\frac{n}{{k}_{w}})}^{\frac{3a}{8}},m\text{'}=\frac{3a}{8},n\text{'}=\frac{13a}{16}$$

the expression for the peak discharge now turns valid for any *a* value, although dependent on the slope of the spillway:

17 $${Q}_{p}=K{\text{'}}^{\frac{8}{8-3a}}{A}^{\frac{8}{8-3a}}{S}^{\frac{13a}{16-6a}}$$

The similar dependence of *Q*_*p*_ on area and erodability relative to Eq. 9 suggests that the relationship between peak discharge and erodability is not changed substantially by the 0D approximation or by the sill vs. spillway choice of formulation.

### Code availability

A copy of the developed C code *spillover* is publicly available on GitHub and here: https://sites.google.com/site/daniggcc/research-interests/lake-overtopping-outburst-megafloods

## Electronic supplementary material

Supplementary Table 1

Supplementary Table 2
